# Supplementary material for: Distinct astrocytic modulatory roles in sensory transmission during sleep, wakefulness, and arousal states in freely moving mice
Source: Nat Commun. 2023 Apr 17;14:2186. doi: 10.1038/s41467-023-37974-z (PMC10110578; doi:10.1038/s41467-023-37974-z)
Supplement: Supplementary file 1 — Supplementary Information [file 41467_2023_37974_MOESM1_ESM.pdf]

Fig. S1

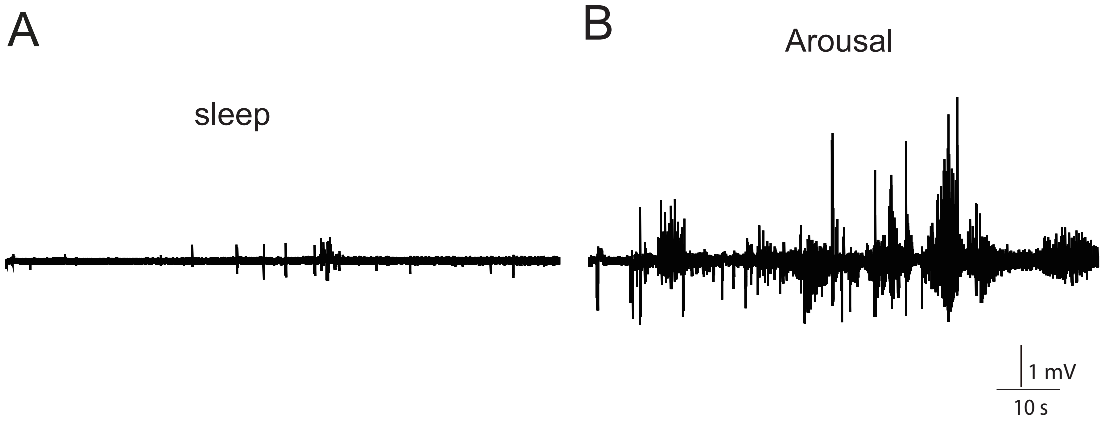

Figure S1. EMG recordings used to monitor behavioral states. A: EMG recordings during sleep. B: EMG recordings during the arousal state.

Fig. S2

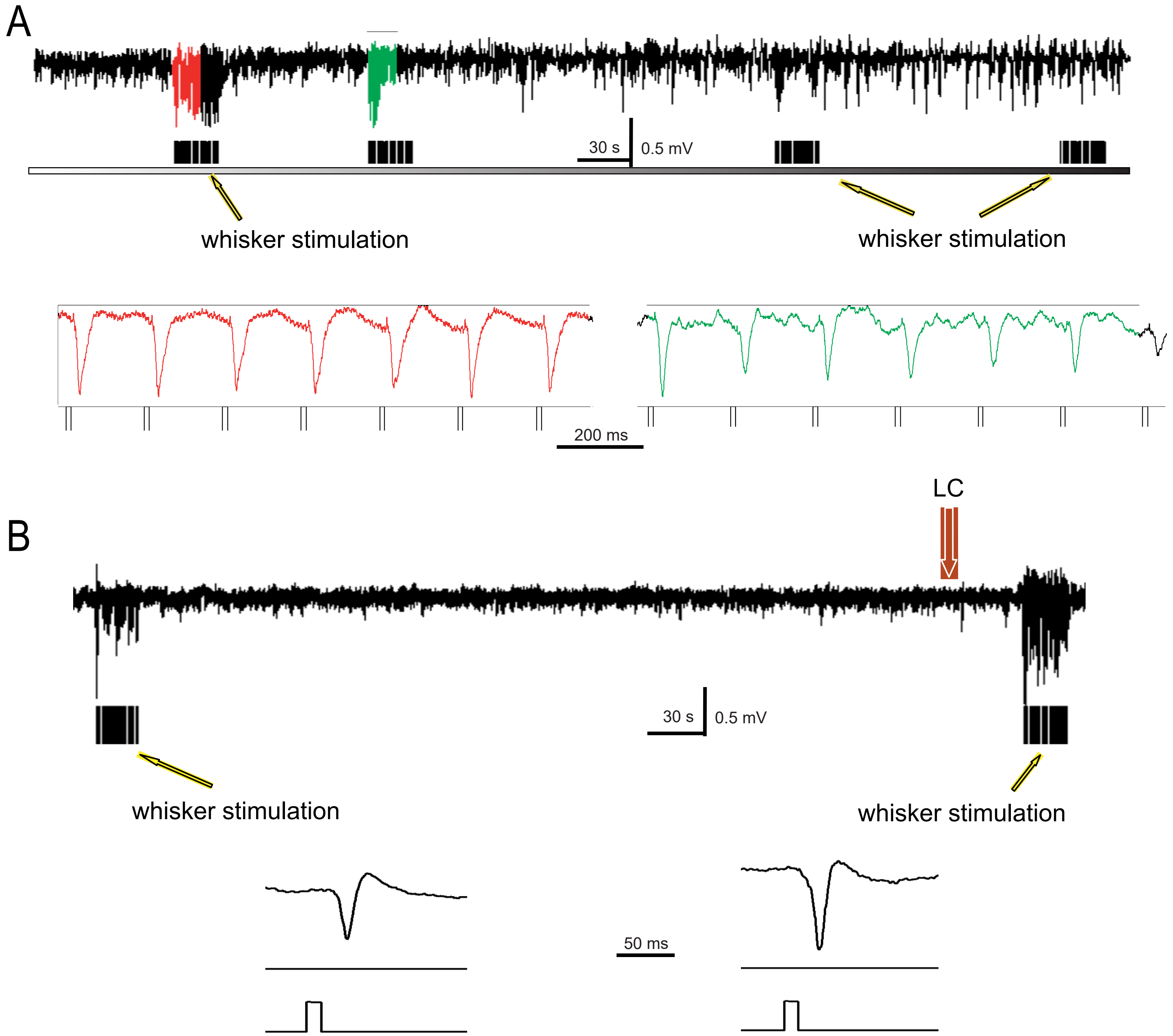

Figure S2. Long-term recording of whisker stimulation-induced EPSPs. A: EPSPs decrease as the animal transitions to sleep. B: EPSPs increase when a sleeping animal is stimulated with LC-NE.

Fig. S3

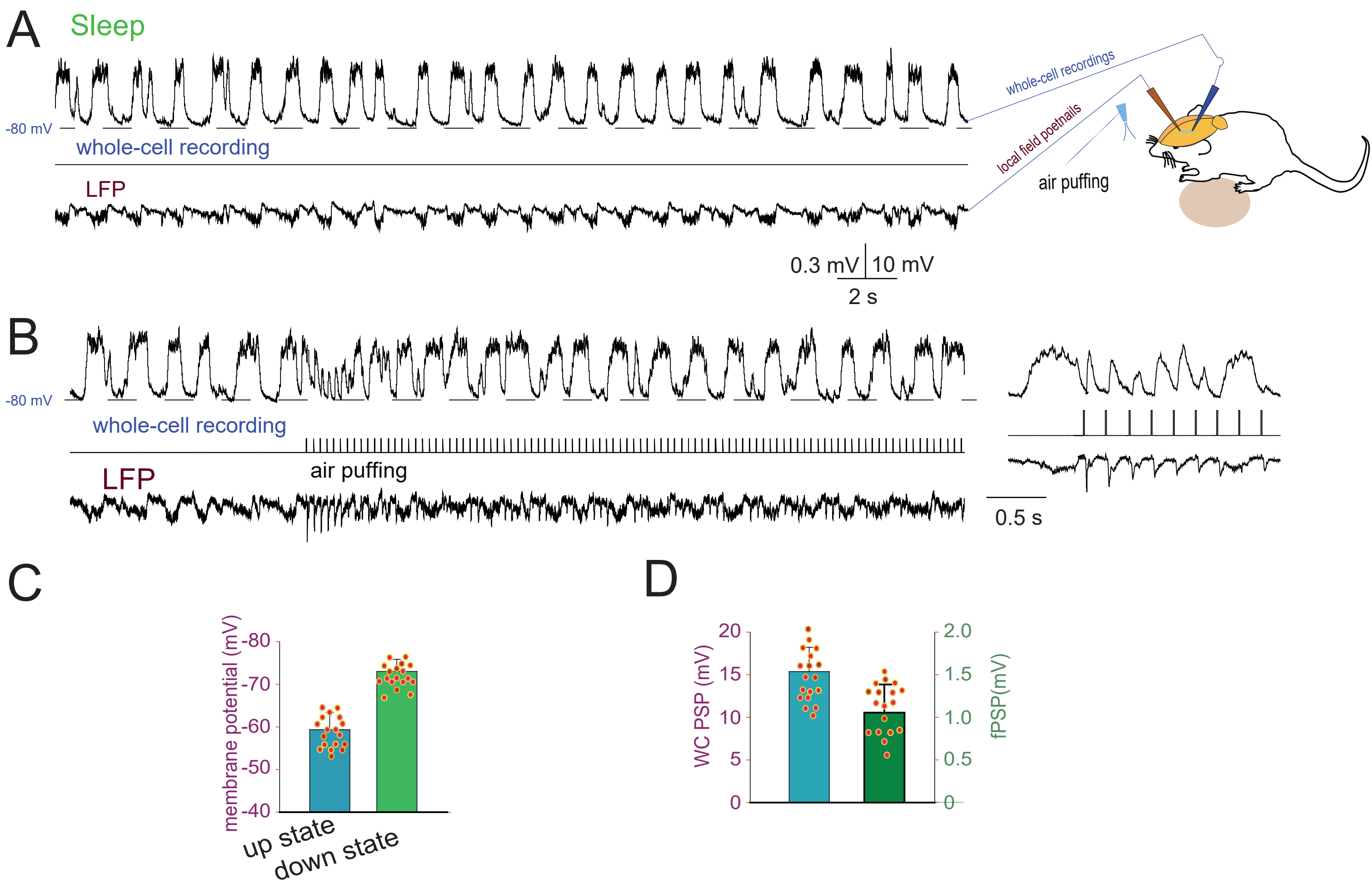

Figure S3. Comparison of membrane potentials and EPSPs for in vivo whole-cell recordings and local field potentials (LFPs). A: Typical recording of membrane potentials during sleep states. The left panel shows the model recordings of *in vivo* whole-cell and LFP recordings. B: Whisker stimulation-induced EPSPs recorded with whole-cell recording and LFP. C: Statistical analysis of membrane potentials for up-states and down-states (n = 18 cells, data are presented as the mean ± SD). D: Comparison of EPSPs recorded with *in vivo* whole-cell recording and LFP recordings (n = 18 mice, data are presented as the mean ± SD).
