# Supplementary material for: Distinct astrocytic modulatory roles in sensory transmission during sleep, wakefulness, and arousal states in freely moving mice
Source: Nat Commun. 2023 Apr 17;14:2186. doi: 10.1038/s41467-023-37974-z (PMC10110578; doi:10.1038/s41467-023-37974-z)
Supplement: Supplementary file 3 — Description of Additional Supplementary Files [file 41467_2023_37974_MOESM3_ESM.pdf]

Supplementary movie legends

Supplementary Movie 1: Small astrocytic Ca2+ imaging during the sleeping state evoked by whisker stimulation at 60 s. The movie shows GCaMP6f-SV40-transfected mice at 3.7 s per series.

Supplementary Movie 2: Large astrocytic Ca2+ signaling during waking states evoked by whisker stimulation at 60 s. The movie shows GCaMP6f-SV40-transfected mice at 2.2 s per series.
