# Supplementary material for: Distinct astrocytic modulatory roles in sensory transmission during sleep, wakefulness, and arousal states in freely moving mice
Source: Nat Commun. 2023 Apr 17;14:2186. doi: 10.1038/s41467-023-37974-z (PMC10110578; doi:10.1038/s41467-023-37974-z)
Supplement: Supplementary file 6 — Reporting Summary [file 41467_2023_37974_MOESM6_ESM.pdf]

nature portfolio

Corresponding author(s): Erxi Wu, Jason Huang

Last updated by author(s): Mar 31, 2023

Reporting Summary

Nature Portfolio wishes to improve the reproducibility of the work that we publish. This form provides structure for consistency and transparency in reporting. For further information on Nature Portfolio policies, see our Editorial Policies and the Editorial Policy Checklist.

Statistics

For all statistical analyses, confirm that the following items are present in the figure legend, table legend, main text, or Methods section.

n/a	Confirmed	
☐	☒	The exact sample size (*n*) for each experimental group/condition, given as a discrete number and unit of measurement
☐	☒	A statement on whether measurements were taken from distinct samples or whether the same sample was measured repeatedly
☐	☒	The statistical test(s) used AND whether they are one- or two-sided<br>*Only common tests should be described solely by name; describe more complex techniques in the Methods section.*
☐	☒	A description of all covariates tested
☐	☒	A description of any assumptions or corrections, such as tests of normality and adjustment for multiple comparisons
☐	☒	A full description of the statistical parameters including central tendency (e.g. means) or other basic estimates (e.g. regression coefficient) AND variation (e.g. standard deviation) or associated estimates of uncertainty (e.g. confidence intervals)
☐	☒	For null hypothesis testing, the test statistic (e.g. *F*, *t*, *r*) with confidence intervals, effect sizes, degrees of freedom and *P* value noted<br>*Give P values as exact values whenever suitable.*
☒	☐	For Bayesian analysis, information on the choice of priors and Markov chain Monte Carlo settings
☒	☐	For hierarchical and complex designs, identification of the appropriate level for tests and full reporting of outcomes
☒	☐	Estimates of effect sizes (e.g. Cohen's *d*, Pearson's *r*), indicating how they were calculated

Our web collection on statistics for biologists contains articles on many of the points above.

Software and code

Policy information about availability of computer code

Data collection	In vivo two-photon imaging and stimulation A custom-built microscope attached to a Tsunami/Millenium laser, (Spectra Physics, Mountain View, CA) and scan box (FV300 Fluoview Software Ver 4.3a, Olympus, Center Valley, PA) was used for 2-photon imaging through a 203 objective (0.9 NA, Olympus). The excitation wavelength was in the range of 800-820 nm. Emission wavelengths were split to detect fluo-4 and AlexaFluor 594 signals as previously described 17. Images of astrocytic Ca2+ signaling were recorded every 2-3 s, which was sufficient to capture evoked responses while limiting laser-induced photodamage at a laser power of <30 mW. Prior to whisker stimulation experiments, anesthesia 0.5 mg/kg D-tubocurarine was injected to prevent small reflex movements that could distort imaging. Direct LC stimulation was applied using a bipolar concentric electrode. Stimulation consisted of a single train of 20--100 pulses (100 Hz, 50 µA, 0.5 ms square pulses). ROIs were extracted from the above-threshold pixels with the fluorescence of the GCaMP or Rhod-2 imaging, and ΔF/F signals were calculated to detect the periods that had multiple ΔF/F peaks above baseline.<br>Codes were not used in this work.
Data analysis	All analyses were performed using SPSS 19 software (IBM) and all tests were two-tailed where significance was achieved at the α = 0.01 level. The data were shown as mean ± S.D. (standard deviation). For independent samples, a t- test (≤ 2 variables) or one-way ANOVA (> 2 variables) was used; for paired samples, a paired t test was used.

For manuscripts utilizing custom algorithms or software that are central to the research but not yet described in published literature, software must be made available to editors and reviewers. We strongly encourage code deposition in a community repository (e.g. GitHub). See the Nature Portfolio guidelines for submitting code & software for further information.

Data

Policy information about availability of data

All manuscripts must include a data availability statement. This statement should provide the following information, where applicable:

- Accession codes, unique identifiers, or web links for publicly available datasets
- A description of any restrictions on data availability
- For clinical datasets or third party data, please ensure that the statement adheres to our policy

All data needed to evaluate the conclusions in the paper are present in the paper and/or the Supplementary Information/Source Data file. Data are also available upon request from the corresponding authors.

Human research participants

Policy information about studies involving human research participants and Sex and Gender in Research.

Reporting on sex and gender	N/A
Population characteristics	N/A
Recruitment	N/A
Ethics oversight	N/A

Note that full information on the approval of the study protocol must also be provided in the manuscript.

Field-specific reporting

Please select the one below that is the best fit for your research. If you are not sure, read the appropriate sections before making your selection.

☒ Life sciences ☐ Behavioural & social sciences ☐ Ecological, evolutionary & environmental sciences

For a reference copy of the document with all sections, see nature.com/documents/nr-reporting-summary-flat.pdf

Life sciences study design

All studies must disclose on these points even when the disclosure is negative.

Sample size	We determined the sample size refer to large amounts of previous literatures and pre-test, as well as sample size calculation for detecting a difference of 0.2 between groups to achieve a minimal of 80% power with type I error of alpha=0.05.
Data exclusions	No data was excluded.
Replication	We used multiple mice in our experiments and the results and standard deviations were within expected ranges.
Randomization	This is not a clinical trial study. Mice used in our study is randomly divided to study groups with no bias.
Blinding	The investigators were blinded to group allocation during the data collection and analyses.

Reporting for specific materials, systems and methods

We require information from authors about some types of materials, experimental systems and methods used in many studies. Here, indicate whether each material, system or method listed is relevant to your study. If you are not sure if a list item applies to your research, read the appropriate section before selecting a response.

March 2021

Materials & experimental systems

n/a	Involved in the study
☒	☐ Antibodies
☒	☐ Eukaryotic cell lines
☒	☐ Palaeontology and archaeology
☐	☒ Animals and other organisms
☒	☐ Clinical data
☒	☐ Dual use research of concern

Methods

n/a	Involved in the study
☒	☐ ChIP-seq
☒	☐ Flow cytometry
☒	☐ MRI-based neuroimaging

Animals and other research organisms

Policy information about studies involving animals; ARRIVE guidelines recommended for reporting animal research, and Sex and Gender in Research

Laboratory animals	Adult (10 weeks old) C57Bl/6 wild-type mice were used (both male and female, Charles River Laboratories). The preparation for mouse experiments was modified from published protocols. Briefly, mice were anesthetized using isoflurane (1.5% mixed with 1-2 L/min O2), head restrained with a custom-made mini-frame and habituated to the restraint over one week in multiple sessions, with a total training duration of 3-4 hours. Mice were housed in an isolation facility with a light/dark cycle of 12/12 hours and provided food and water ad libitum.
Wild animals	No wild animals are used in this study.
Reporting on sex	Both sexes
Field-collected samples	No field-collected samples are used in this study.
Ethics oversight	All animal procedures followed the National Institute of Health guidelines and were approved by the Institution of Animal Care and Use Committee at the University of Rochester, and the Institution of Animal Care and Use Committee at Sichuan Normal University.

Note that full information on the approval of the study protocol must also be provided in the manuscript.
